# The features of high-risk human papillomavirus infection in different female genital sites and impacts on HPV-based cervical cancer screening

**DOI:** 10.1186/s12985-023-02073-4

**Published:** 2023-06-06

**Authors:** Tingyuan Li, Simiao Chen, Xinyue Li, Zeni Wu, Yuqian Zhao, Jianfeng Cui, Bin Liu, Feng Chen, Xun Zhang, Youlin Qiao, Wen Chen

**Affiliations:** 1 Department of Cancer Epidemiology, National Cancer Center/National Clinical Research Center for Cancer/Cancer Hospital, Chinese Academy of Medical Sciences and Peking Union Medical College, Beijing, 100021 China; 2 Sichuan Clinical Research Center for Cancer, Sichuan Cancer Hospital & Institute, Sichuan Cancer Center, Affiliated Cancer Hospital of University of Electronic Science and Technology of China, Chengdu, 610041 China; 3 College of Life Sciences, Institute of Life Science and Green Development, Hebei University, Baoding, 071000 China; 4 Department of pathology, National Cancer Center/National Clinical Research Center for Cancer/Cancer Hospital, Chinese Academy of Medical Sciences and Peking Union Medical College, Beijing, 100021 China; 5 Center for Global Health, School of Population Medicine and Public Health, Chinese Academy of Medical Sciences & Peking Union Medical College, Beijing, 100730 China

**Keywords:** Human papillomavirus, Female genital tract, Viral load, Cervical cancer

## Abstract

**Background:**

The causal role of high-risk Human papillomavirus (HR-HPV) in the pathogenesis of anogenital cancers is well established. In contrast, information on HR-HPV distribution of continuous anatomic sites within the female genital tract is limited, and the impact of sample type on the clinical performance in HPV-based cervical cancer screening warrants investigation.

**Methods:**

A total of 2,646 Chinese women were enrolled in the study from May 2006 to April 2007. We analyzed the infection features by infection status and pathological diagnoses of 489 women with complete HR-HPV type and viral load data on the cervix, upper vagina, lower vagina, and perineum samples. Additionally, we assessed the clinical performance for detecting high-grade cervical intraepithelial neoplasia of grade two or worse (≥ CIN2) among these four types of samples.

**Results:**

HR-HPV positivity rate was lower in the cervix (51.53%) and perineum (55.83%), higher in the upper (65.64%) and lower vagina (64.42%), and increased with the severity of cervical histological lesions (all P<0.001). Single infection was more dominant than multiple infections at each anatomic site of the female genital tract. The proportion of single HR-HPV infection decreased successively from the cervix (67.05%) to the perineum (50.00%) (*P*_*trend*_=0.019) in cervical intraepithelial neoplasia grade 1 (CIN1) and was higher in samples of the cervix (85.11%) and perineum (72.34%) in ≥ CIN2. In addition, the highest viral load was observed in the cervix compared to the other three sites. The overall agreement of the cervical and perineum samples was 79.35% and increased continuously from normal (76.55%) to ≥ CIN2 (91.49%). As for the detection of ≥ CIN2, the sensitivity was 100.00%, 97.87%, 95.74%, and 91.49% for the cervix, upper vagina, lower vagina, and perineum samples, respectively.

**Conclusions:**

Single HR-HPV infection predominated throughout the female genital tract, but the viral load was lower compared to multiple HR-HPV infections. Despite the decreasing viral load from cervix to perineum, the clinical performance for detecting ≥ CIN2 of the perineum sample was comparable to that of the cervix.

**Supplementary Information:**

The online version contains supplementary material available at 10.1186/s12985-023-02073-4.

## Introduction

Genital Human papillomavirus (HPV) infection causes almost all cervical cancer and is associated with other anogenital cancers. Nearly 4.5% of all cancers worldwide (630,000 new cancer cases per year) are mainly due to HPV infection: 8.6% in women and 0.8% in men [1]. Of the over 200 types of HPV, about 40 types can infect the epithelium of the anogenital tract or other mucosae [2, 3], and at least 13 types (16, 18, 31, 33, 35, 39, 45, 51, 52, 56, 58, 59, and 68) are highly carcinogenic to humans [4]. These carcinogenic (or high-risk [HR]) HPV infections are frequent after sexual contact. Although most HPV infections are asymptomatic and generally cleared within two years after sexual transmission, persistent high-risk HPV infections are at the greatest risk for starting the oncogenic process from a preneoplastic lesion to invasive cancer [5]. HPV testing has become a crucial part of cervical cancer screening and is an effective primary screening method in cervical cancer prevention strategies [6].

Within the past two decades, there has been an increased interest in the clinical validity of various sampling methods in cervical cancer screening, such as urine and vaginal HPV self-sampling [7, 8]. These sampling methods can increase the screening coverage of a targeted population [9] and maintain good clinical performance for detecting cervical lesions [10]. Due to their low cost, non-invasiveness, and acceptability, vaginal and urine self-collected samples provide an alternative way to solve the problem of poor screening uptake. However, further evaluation on these sampling methods and the comparison with the “gold standard” cervical sample is necessary [11].

The distal vagina, clitoris, and urethra are integrated entities covered superficially by the vulval skin and its epithelial features [12]. As a result, urine HPV might be affected by the HPV viral load in the perineum or genital tract. The infection features at other anatomic sites of the female genital tract beyond the cervix (e.g., the vagina and perineum) may explain the natural history of HR-HPV infection in the female reproductive tract, help control the HR-HPV infection, and provide improved HPV detection in urine.

In a previous study of HR-HPV genotype distribution in the female genital tract, we found concordance between the cervix and other genital sites [13]. However, infection features of HR-HPV throughout the female genital tract, including viral load and status of single/multiple infections, are poorly understood, especially for the perineum.

Here, we describe the infection status and viral load of HR-HPV in the cervix, upper vagina, lower vagina, and perineum to provide more evidence for the natural history of HR-HPV infection throughout the female genital tract. In addition, we evaluate the concordance of HPV detection for cervical intraepithelial neoplasia of grade two or worse (≥ CIN2) using different samples.

## Materials and methods

### Study population

**Fig. 1:**
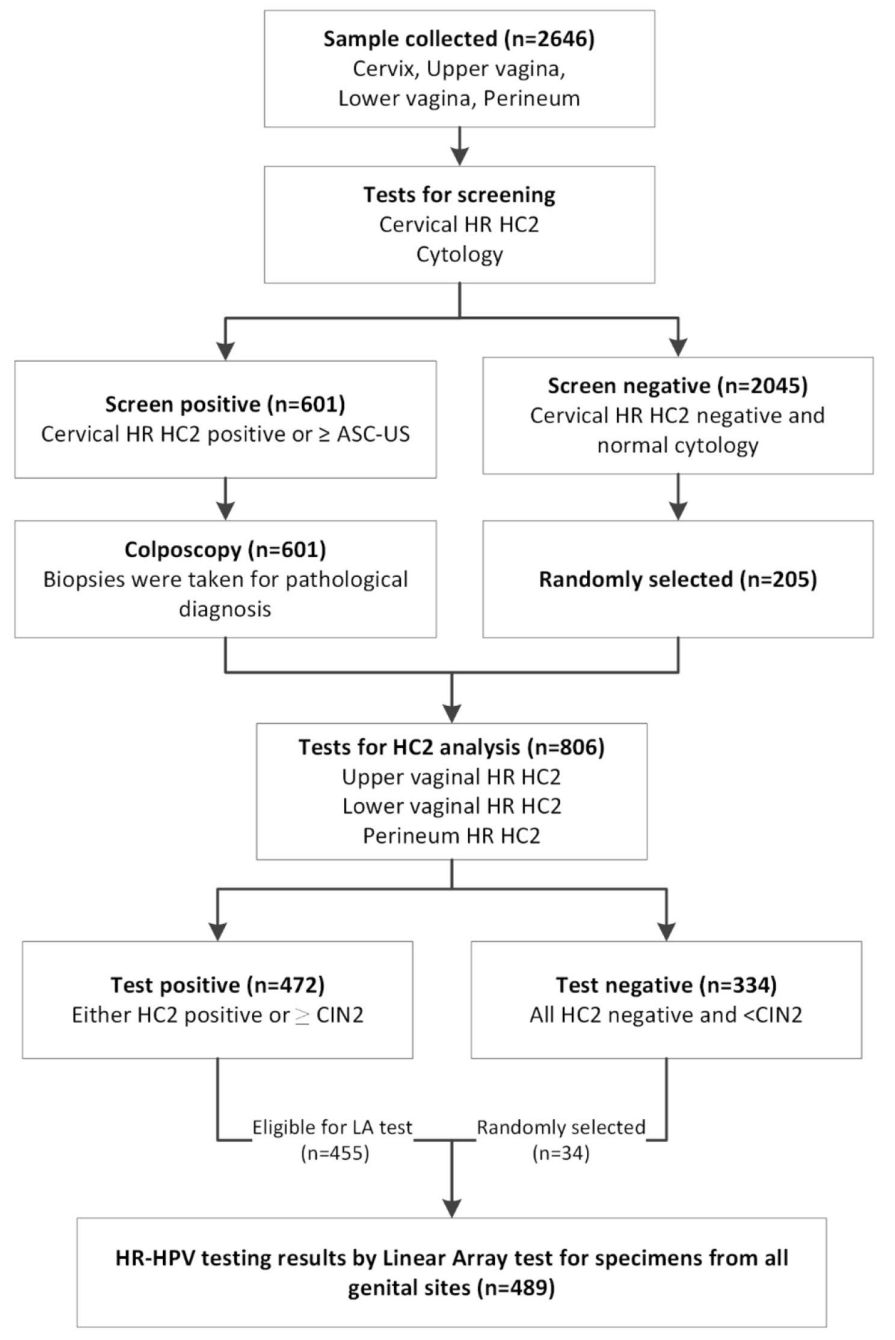
Flowchart of the study

This research was part of a multi-center, population-based study of cervical cancer screening in China’s rural areas (Shanxi Province Cervical Cancer Screening Study III, SPOCCS III). A total of 2,646 women were enrolled in the study from May 2006 to April 2007. The inclusion and exclusion criteria and study procedures have been described previously [13, 14]. Briefly, specimens were collected by local gynecologists sequentially from 4 anatomic sites of the female genital tract: perineum, lower vagina, upper vagina, and cervix. Hybrid Capture2 High-Risk HPV DNA (HC2 HR-HPV) test and Linear Array were adopted as the HPV detection methods. Women with cervical HR-HPV positive tested by HC2 HR-HPV test or abnormal cytology received a colposcopy test, and the samples from the other three sites were further tested by HC2 HR-HPV test and Linear Array. Approximately 10% of screen-negative women were randomly selected and completed the screening workflow. Ultimately, 489 women with complete HPV type and viral load data on the cervix, upper vagina, lower vagina, and perineum samples were included in the final analysis (Fig. 1). This study was approved by the institutional review board (IRB) of the Cleveland Clinic and the Cancer Institute/Hospital of the Chinese Academy of Medical Sciences (CICAMS).

### Pathology diagnosis

Histological slides were diagnosed by local pathologists, with a quality control sample of 35 slides (15 CIN1, 10 CIN2, and 10 CIN3) reread by a panel of three pathologists. The original histological interpretation by the local pathologists was used for data analysis, and screen-negative women were defined as pathology-negative.

### Detection of HPV viral load and genotyping

HR-HPV viral load was estimated using the signal strength of relative light unit /cutoff ratio (RLU/CO) detected by Hybrid Capture2 High-Risk HPV DNA test, and genotypes of HPV were further identified by Linear Array (Roche, Pleasanton, CA) assay. The HC2 HR-HPV test was a signal-amplified hybridization microplate-based assay and can detect 13 high-risk genotypes, including HPV16, 18, 31, 33, 35, 39, 45, 51, 52, 56, 58, 59, and 68 semi-quantitatively. RLU/CO ≥ 1 was defined as positive for the HC2 HR-HPV test [13].

Linear Array is a HPV genotyping test and can detect up to 37 individual HPV genotypes simultaneously (i.e., genotypes 6, 11, 16, 18, 26, 31, 33, 35, 39, 40, 42, 45, 51, 52, 53, 54, 55, 56, 58, 59, 61, 62, 64, 66, 67, 68, 69, 70, 71, 72, 73, 81, 82, 83, 84, IS39, and CP6108) [15]. Linear Array results were considered HR-HPV positive only if one of the 13 HR-HPV types targeted by HC2 was detected. All detections were performed according to the manufacturer’s instructions.

### Statistical analysis

Specimens with RLU/CO of one by HC2 HR-HPV test were supposed to contain one pg/ml of viral load. Therefore, the signal strength of HC2 HR-HPV was used to describe the viral load of each sample. Infection rate of HR-HPV was calculated based on the Linear Array test result. The *single infection* was defined as an infection of only one of the 13 HR-HPV types, whereas *multiple infections* were defined as co-infections with two or more of the 13 HR-HPV types. Comparison of infection rates of HR-HPV for different anatomic sites of the female genital tract was analyzed using Chi-square tests. Chi-square trend tests were used to investigate the HR-HPV infection variation tendency from the cervix to the perineum and from normal to high-grade cervical lesions. ANOVA tests were conducted to estimate the viral load variation in different anatomic sites of the female genital tract and pathological diagnoses. Data were analyzed using R software (V4.0.3), and P < 0.05 (two-sided) was considered statistically significant.

## Results

### HR-HPV positivity rate by infection status and pathological diagnoses

**Fig. 2:**
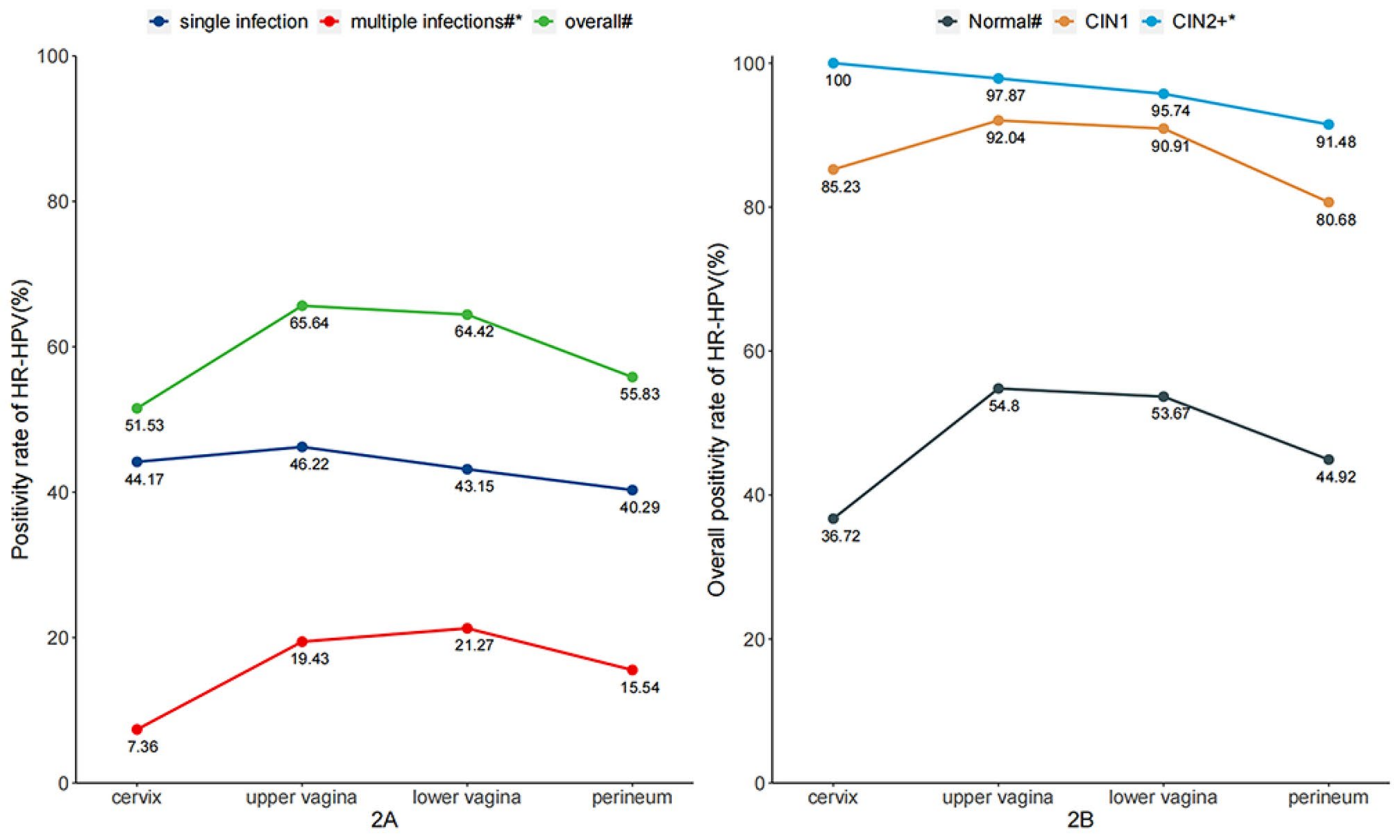
The comparison of HR-HPV positive rate by infection status and pathological diagnoses. Note: #, statistical difference by Chi-square test; *, statistical difference by Chi-square trend test

Among the 489 subjects, there were 47 ≥ CIN2, 88 CIN1, and 354 with negative pathology. The overall HR-HPV positivity rate was significantly different among female genital sites (P < 0.001, Fig. 2A), with lower percentage in cervix (n = 252, 51.53%) and perineum (n = 273, 55.83%) compared to upper (n = 321, 65.64%) and lower vagina (n = 315, 64.42%). The positivity rate of HR-HPV single infection showed no significance among different anatomic genital sites (P = 0.303, Fig. 2A). However, the prevalence of multiple infections demonstrated significant differences among anatomic genital sites (P < 0.001), with increasing tendency from the cervix (n = 36, 7.36%) to the perineum (n = 76, 15.54%) (P_*trend*_<0.001, Fig. 2A). In addition, at each anatomic site of the female genital tract, the prevalence of single infection was higher than that of multiple infections (Fig. 2A). The positivity rates of HR-HPV increased with the elevation of cervical histological lesions at each site of the female genital tract (Fig. 2B). Positivity rates of HR-HPV showed different tendencies from the cervix to perineum in women with different histological diagnoses. In women diagnosed with ≥ CIN2, the positivity rate decreased successively from the cervix (n = 47, 100%) to the perineum (n = 43, 91.48%) (P_*trend*_*=0.026)*, while in women diagnosed with CIN1, higher positivity rates were found in the upper (n = 81, 92.04%) and lower vagina (n = 80, 90.91%) compared to cervix (n = 75, 85.23%) and perineum (n = 71, 80.68%) (Fig. 2B).

### Trends in single or multiple infections stratified by pathological diagnoses

**Fig. 3:**
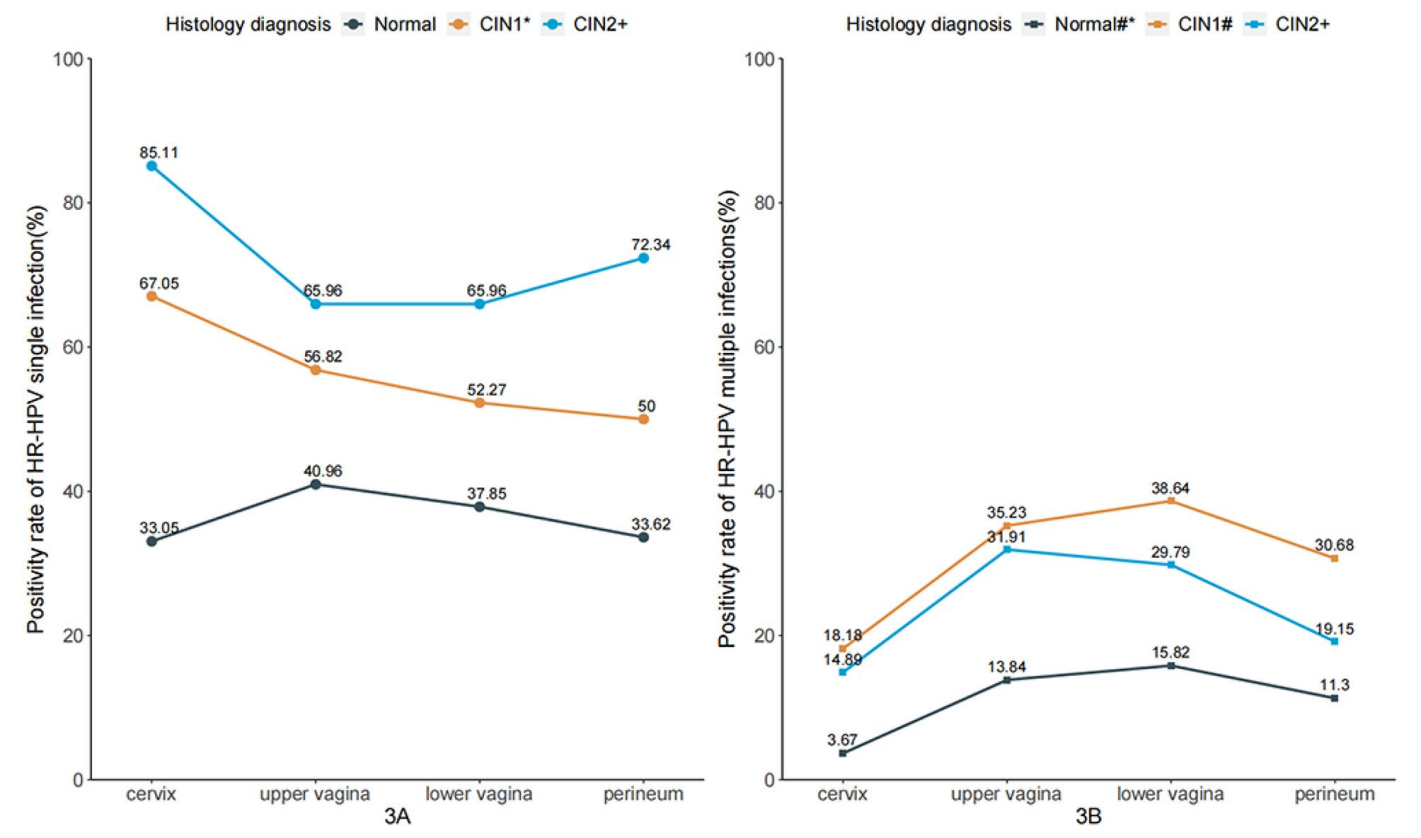
Prevalence of HR-HPV single and multiple infections in different anatomic sites by pathological diagnoses. Note: #, statistical difference by Chi-square test; *, statistical difference by Chi-square trend test

There were significant differences in the positivity rate of HPV when focusing on single and multiple infection rates in different anatomic sites by pathological diagnoses (Fig. 3). The positivity rate of single HR-HPV infection decreased successively from the cervix (n = 59, 67.05%) to the perineum (n = 44, 50.00%) (P_*trend*_=0.019) in women with CIN1 (Fig. 3A). In women with ≥ CIN2, the single HR-HPV infection rate was higher in samples from the cervix (n = 40, 85.11%) and perineum (n = 34, 72.34%); however, in women with a normal cervix, higher positivity rates were found in samples from the upper (n = 145, 40.96%) and lower vagina (n = 134, 37.85%) (Fig. 3A). As for multiple infections (Fig. 3B), the HR-HPV positivity rate increased successively from the cervix to the perineum, which was statistically different among the four sites of the female genital tract for women with normal or CIN1 cervix (P < 0.001). In women with ≥ CIN2, the multiple infection rates differed by anatomic sites, but no statistical difference was found (P = 0.156, Fig. 3B). In addition, in different cervical pathological diagnoses, the single HR-HPV infection was more dominant than multiple infections at each anatomic site of the female genital tract (Fig. 3).

### Variation of HR-HPV viral load by infection status and pathological diagnoses

**Fig. 4:**
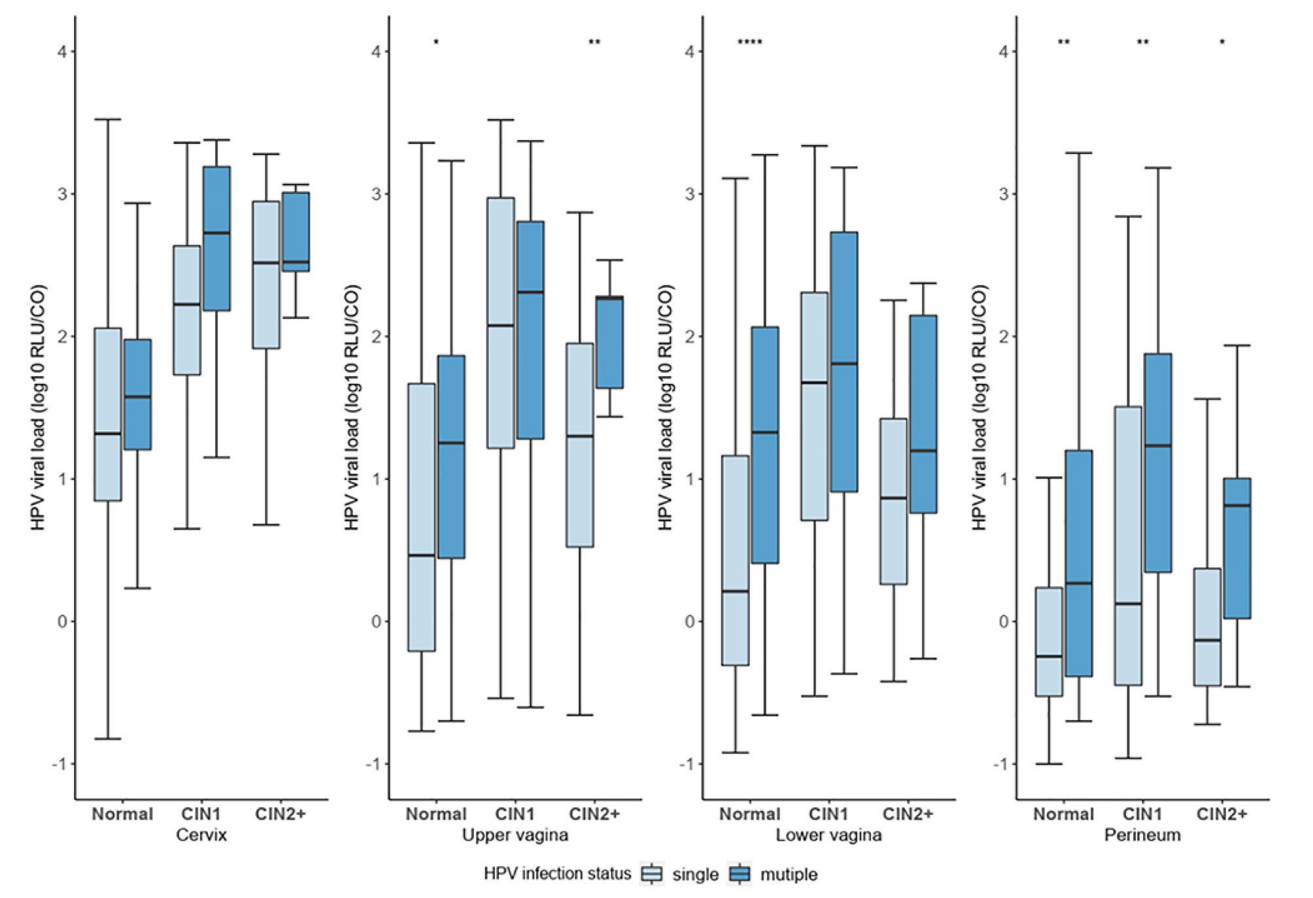
The variation of HR-HPV viral load by infection status and pathological diagnoses. Note: ****,<0.0001; **,<0.01; *,<0.05

Viral load in the cervix was higher than the other three anatomical sites, regardless of the infection status or pathological diagnoses (Fig. 4), with viral load in the perineum being the lowest. At each site of the female genital tract, the viral load of the single infection was lower, on average, than that of multiple infections (Figure S1). In the upper vagina, the viral load difference between single and multiple infections was significant in women with ≥ CIN2 (P = 0.007) or normal cervix (P = 0.040) but not in women with CIN1 (P = 0.891). In the lower vagina, the viral load of different infection statuses was statistically different in the normal cervix (P<0.001) but not statistically significant in CIN1(P = 0.491) or ≥ CIN2 (P = 0.144). At the perineum, single infection viral load was significantly lower than multiple infections across all pathological diagnoses (P<0.05, respectively).

### Agreement of HR-HPV between cervix and perineum sample detected by Linear array

Table 1 shows the agreement of HR-HPV between the cervix and perineum samples detected by Linear Array. Overall, the positive agreement rate was 67.73% (95%CI: 63.59–71.87%), and the overall agreement rate was 79.35% (95%CI: 75.76–82.94%). When considering pathological diagnoses, positive agreement and overall agreement increased with the elevation of cervical lesions. From normal cervix to ≥ CIN2, positive agreement increased from 55.38% (95%CI: 50.20-60.56%) to 91.49% (95%CI: 83.51–99.47%) (P_*trend*_<0.001), and overall agreement increased from 76.55% (95%CI: 72.14–80.96%) to 91.49% (95%CI: 83.51–99.47%) (P_*trend*_=0.026).

**Table 1:** The agreement between cervix sample and perineum sample according to Linear Array

Pathological diagnoses	Positive agreement (%; 95% CI)	P _*trend*_	Overall agreement (%; 95% CI)	P _*trend*_
Overall population (N = 489)	67.73 (63.59–71.87)	–	79.35 (75.76–82.94)	–
Normal (n = 354)	55.38 (50.20-60.56)	< 0.001	76.55 (72.14–80.96)	0.026
CIN1 (n = 88)	82.50 (74.56–90.44)		84.09 (76.45–91.73)	
≥CIN2 (n = 47)	91.49 (83.51–99.47)		91.49 (83.51–99.47)	

### Clinical performance for the detection of ≥ CIN2 by different samples

Clinical performance for detecting ≥ CIN2 using different samples is depicted in Table 2. Detection sensitivity was not statistically different (P = 0.158), but showed decreasing trend from cervix to perineum (P_*trend*_=0.026), which was 100.00% (95%CI: 98.40–100.00%) in cervix samples, 97.87% (95%CI: 88.89–99.62%) in upper vagina samples, 95.74% ( 95%CI: 85.75–98.83) in lower vagina samples, and 91.49% in perineum samples (95%CI: 80.07–96.64%). In addition, the specificity of upper (37.78%, 95%CI: 33.39–42.39%) and lower vagina (38.91%, 95%CI: 34.48–43.54%) samples were silghtly lower compared to cervical sample (P < 0.05). The area under the curve (AUC) of cervix samples and perineum samples were 0.768 (95%CI: 0.720–0.817) and 0.697 (95%CI: 0.632–0.763), respectively.

**Table 2:** The clinical performance for ≥ CIN2 detection of HPV detected in cervix, upper vagina, lower vagina, and perineum samples

Sample sources	Sensitivity (%; 95% CI)	Specificity (%; 95% CI)	PPV (%; 95% CI)	AUC
Cervix	100.00(98.40–100.00)	53.62(48.96–58.22)	18.65(14.33–23.92)	0.768(0.720–0.817)
Upper vagina	97.87(88.89–99.62)	37.78(33.39–42.39)*	14.33(10.92–18.59)	0.678(0.615–0.741)
Lower vagina	95.74(85.75–98.83)	38.91(34.48–43.54)*	14.29(10.85–18.58)	0.673(0.608–0.738)
Perineum	91.49(80.07–96.64)*	47.96(43.34–52.62)	15.75(11.91–20.54)	0.697(0.632–0.763)

Note: * indicates a statistically significant difference between this site and cervix (*p* value < 0.05)

## Discussion

Cervical cancer screening strategies should consider accuracy, accessibility, and acceptability, especially for women living in low- and middle-income areas [16]. Studying the features of HR-HPV infection throughout the female genital tract will provide an epidemiological basis for seeking the most cost-effective strategies to prevent and control HR-HPV infection [17]. In this research, we analyzed the infection status and viral load of HR-HPV and evaluated the clinical performance for detecting ≥ CIN2 at four anatomic sites of the female genital tract in a population of Chinese women. To our knowledge, this is the first study that simultaneously explores HR-HPV prevalence and viral load variation encompassing everything from the cervix to the perineum.

Our data indicated that the overall HR-HPV prevalence in the perineum is comparable to that of the cervix, while higher infection rates were found in the upper and lower vagina, perhaps due to the high prevalence of multiple HR-HPV infections. As for infection status, previous studies showed that single HR-HPV infection predominated in the HPV-positive population [18–20], and a similar phenomenon was observed in this current study. Additionally, although the prevalence of single HR-HPV infection was decreasing from the cervix to the perineum (not statistically significant), for the upper vagina, lower vagina, and perineum, single HR-HPV infection was also the dominant status, regardless of the pathological diagnoses. On the other hand, the infection rate for multiple HR-HPV infections increased from the cervix to the perineum.

The high prevalence of multiple HR-HPV infections might contribute to higher viral load [21], one of the major determinants of HPV persistence [2]. Therefore, despite the predominant prevalence of single HR-HPV infection at all sites of the genital tract, its viral load was lower than that of multiple HR-HPV infections. In addition, since the multiple infections were more common in the upper and lower vagina, compared with the cervix or the perineum, this might indicate a higher possibility of HPV persistence in these sites, but how it affects cervical infections requires further study. Moreover, the viral load of HR-HPV increased sequentially from the perineum to the cervix in both single and multiple-infection status, which might explain the higher risk of HPV infection resulting in developing cervical cancer than vaginal or vulvar cancer. The low viral load of the perineum also suggested that the limit of detection (LOD) of an HPV detection technology should be taken into consideration, and polymerase chain reaction (PCR)-based techniques seem to be more appropriate for HPV detection of perineum or urine samples, given the ability to identify viral DNA at low levels.

Similar to the cervix [22, 23], HR-HPV positivity rates in the upper vagina, lower vagina, or perineum increased with the severity of cervical lesions. However, the variation of HR-HPV viral load was different. Only in the cervix was the HR-HPV viral load positively-changed with the cervical lesions; in the upper vagina, lower vagina, or perineum, higher HR-HPV viral load was found in CIN1. Since high viral loads are associated with infection persistence [24], more prospective studies are needed to determine whether the cervix HPV infection status will be affected by the viral load in the upper and lower vagina or not.

The agreement of HR-HPV between the cervix and the perineum samples was good and increased with the elevation of cervical lesions, which suggests that HPV in the perineum is a good reflection of the infection status of the cervix, especially in women with ≥ CIN2. Although the sensitivity of the perineum sample was lower than that of the cervix, the upper vagina, or the lower vagina, it was still as high as 91%. At the same time, the specificity and PPV of the perineum sample were comparable to the cervix sample and higher than that of the upper and lower vagina sample. In this study, specificity was lower compared to other studies [25], which might be due to the small number of negative tests in the population. However, the PCR-based HPV detection using perineum samples proved consistent with cervical samples, supporting the feasibility of HPV detection in urine.

Several limitations should be mentioned in this study. Firstly, HPV viral load was defined as the signal strength tested by the HC2 HR-HPV test, which might be affected by the number of heavily-infected cells sampled. This study could not measure the bias in sampling collection. However, viral load measured by HC2 RLU/CO was found to correlate well with that by real-time polymerase chain reaction [26]. In addition, this study lacked prospective follow-up, and information on the correlation between persistent HR-HPV infection status in different genital sites and the risk of cervical or genital lesions is limited.

## Conclusions

This study updated epidemiologic evidence on HR-HPV infection and viral load variation among the cervix, the upper vagina, the lower vagina, and the perineum. We observed that the single HR-HPV infection predominated throughout the female genital tract, but the viral load of single HR-HPV infection was lower than multiple infections at any site of the female genital tract. Then, we noted that despite the variation in viral loads, the clinical performance of the perineum sample was comparable to that of the cervix sample, and PCR-based techniques were recommended for HPV detection of perineum or urine samples. Lastly, more in-depth studies will need to be conducted to determine whether the high viral load of the upper and lower vagina in CIN1 affects the persistent HR-HPV infection of the cervix.

## Electronic supplementary material

Below is the link to the electronic supplementary material.

Supplementary Material 1

## Data Availability

The datasets used or analyzed during the current study are available from the corresponding author on reasonable request.

## Abbreviations

HR-HPV: High risk Human papillomavirus
≥CIN2: High-grade cervical intraepithelial neoplasia of grade two or worse
CIN1: Cervical intraepithelial neoplasia grade 1
HC2 HR-HPV: Hybrid Capture2 High-Risk HPV DNA test
IRB: Institutional review board
CICAMS: Cancer Institute/Hospital of the Chinese Academy of Medical Sciences
RLU/CO: Relative light unit /cutoff ratio
AUC: The area under the curve
CI: Confidence interval
LOD: Limit of detection
PCR: Polymerase chain reaction
PPV: Positive predictive value
