# Supplementary figures and images for: The features of high-risk human papillomavirus infection in different female genital sites and impacts on HPV-based cervical cancer screening
Source: Virol J. 2023 Jun 6;20:116. doi: 10.1186/s12985-023-02073-4 (PMC10246399; doi:10.1186/s12985-023-02073-4)

Figure S1


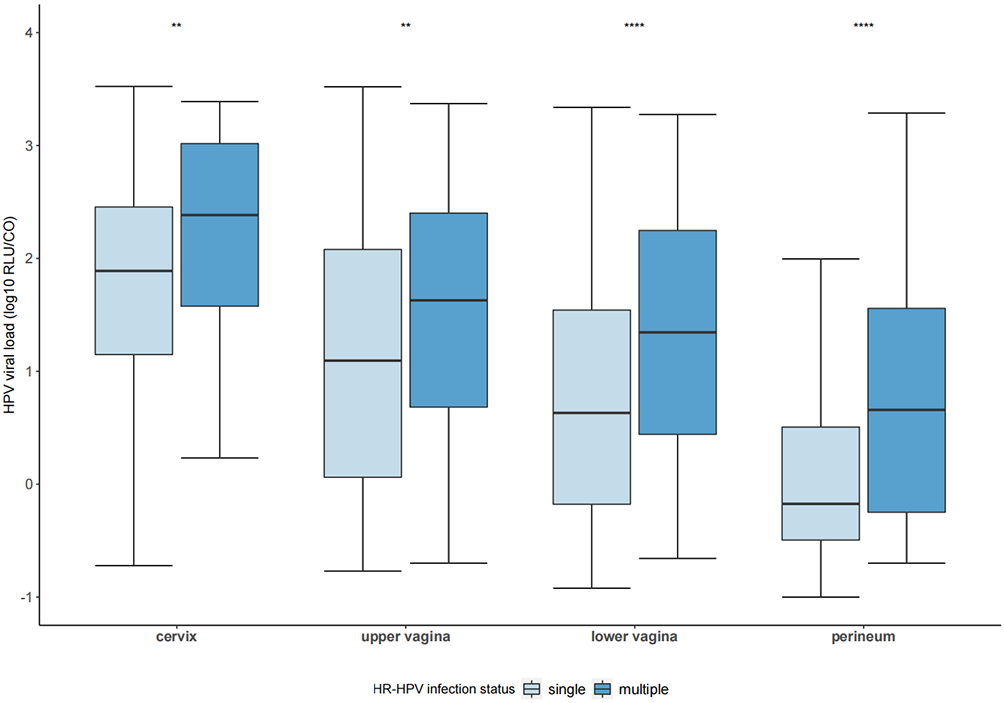

Supplement: Supplementary file 1 — Supplementary Material 1 [file 12985_2023_2073_MOESM1_ESM.docx]
